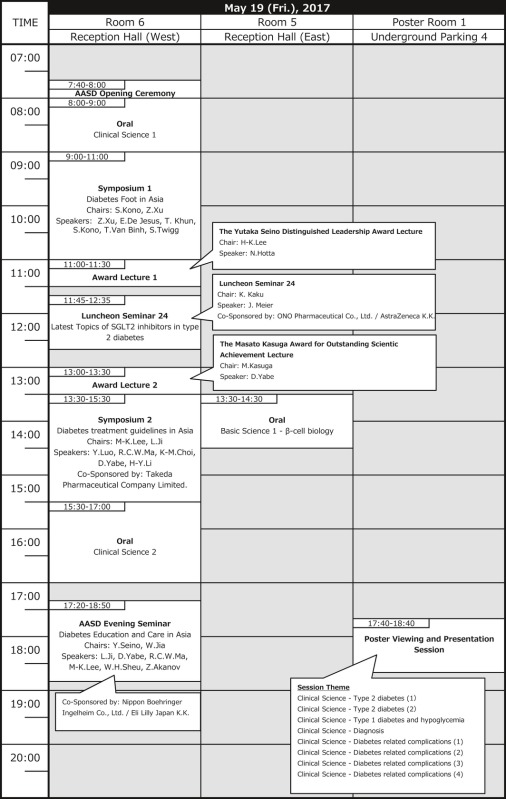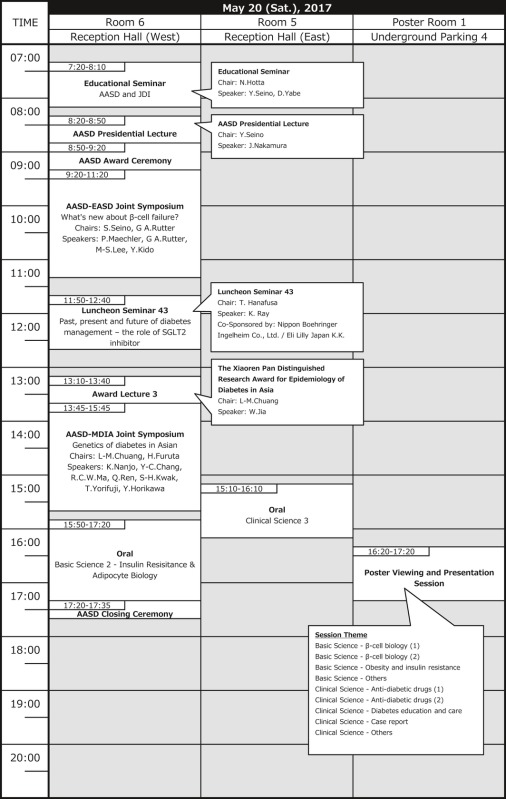# Timetable

**DOI:** 10.1111/jdi.12684

**Published:** 2017-05-05